# Microbiological Characteristics of the Gastrointestinal Tracts of Jersey and Holstein Cows

**DOI:** 10.3390/ani14213137

**Published:** 2024-11-01

**Authors:** Lei Wang, Kai Wang, Lirong Hu, Hanpeng Luo, Shangzhen Huang, Hailiang Zhang, Yao Chang, Dengke Liu, Gang Guo, Xixia Huang, Qing Xu, Yachun Wang

**Affiliations:** 1 College of Animal Science and Technology, China Agricultural University, Beijing 100193, China; wledu2016@163.com (L.W.); wangkai7kkw@gmail.com (K.W.); lironghu92@gmail.com (L.H.); luohanpeng@cau.edu.cn (H.L.); hsz19980225@163.com (S.H.); zhl108@cau.edu.cn (H.Z.); yaoooochang@cau.edu.cn (Y.C.); 2 College of Animal Science and Technology, Xinjiang Agricultural University, Urumqi 830052, China; au-huangxixia@163.com; 3 College of Life Sciences and Bioengineering, Beijing Jiaotong University, Haidian District, Beijing 100044, China; 4 Beijing Sunlon Livestock Development Company Limited, Beijing 100029, China; 13910356698@163.com (D.L.); guogang2180@126.com (G.G.)

**Keywords:** Jersey cows, Holstein cows, gastrointestinal microbiome

## Abstract

The gastrointestinal bacterial microbiota is essential for maintaining the health of dairy cows and ensuring their production potential, and it may also help explain the breed-related phenotypic differences. Therefore, investigating the differences in gastrointestinal bacterial microbiota between breeds is critical for deciphering the mechanisms behind these differences and exploring the potential for improving milk production by regulating the gastrointestinal bacterial microbiota. This study holistically examined the differences between rumen and hindgut bacterial microbiota in a large cohort of two breeds of dairy cows, comprising 184 Jersey cows and 165 Holstein cows. Significant distinctions were identified between the rumen and hindgut bacterial microbiota of dairy cows, with these differences being consistent across breeds. A total of 20 breed-differentiated microorganisms, comprising 14 rumen microorganisms and 6 hindgut microorganisms, were screened, which may be the primary drivers of the observed differences in lactation performance between Jersey and Holstein cows. The present study revealed the spatial heterogeneity of the gastrointestinal bacterial microbiota of Jersey and Holstein cows and identified microbial biomarkers of different breeds. These findings enhance our understanding of the differences in the gastrointestinal bacterial microbiota between Jersey and Holstein cows and may provide useful information for optimizing the composition of the intestinal bacterial microbiota of the two breeds of dairy cows.

## Simple Summary

Jersey cattle is a breed of cow that originated in Jersey, England, and is renowned for its superior milk production capabilities. This breed is known to have a superior milk composition quality to that of Holstein cows, although its milk yield is inferior. However, the gastrointestinal bacterial microbiota of Jersey and Holstein cows remains poorly characterized. To elucidate the mechanisms behind the differences in their lactation performance and to explore the potential to improve milk yield through the modulation of the gastrointestinal bacterial microbiota, it is essential to understand the structure of the gastrointestinal bacterial microbiota of the two breeds under the same feeding environment. In this study, we employed 16S rRNA gene sequencing to investigate the gastrointestinal bacterial microbiota of Jersey and Holstein cows. This study revealed that the bacterial diversity of the hindgut bacterial microbiota was lower than that of the rumen bacterial microbiota in both breeds. Additionally, we observed that the gastrointestinal regions of both Jersey and Holstein cattle breeds had specific bacteria. In summary, there was spatial heterogeneity in the bacterial microbiota between gastrointestinal tracts, and specific microbial biomarkers were identified between breeds.

## 1. Introduction

Ruminants have complex microecosystems in their gastrointestinal tract (GIT) which plays a crucial role in feed degradation, metabolism, tissue development, and immune regulation of the host [1]. Each separate region in the GIT harbors different microbial communities due to variations in pH, nutrient availability, and other physiological and biochemical factors [1,2,3,4,5]. Due to the feasibility and convenience of live sampling, research on rumen and hindgut microorganisms is of significant importance for understanding the microbiota of ruminants. It was observed that the microbial community diversity in the rumen is generally higher than that in the hindgut. Rumen bacteria predominantly consist of *Firmicutes* and *Bacteroidetes*, with key genera including *Fibrobacter*, *Ruminococcus*, *Prevotella*, and *Butyrivibrio*, which primarily ferment complex plant materials such as cellulose and hemicellulose into volatile fatty acids (VFAs), hydrogen and carbon dioxide [1]. The cecum, colon and rectum, collectively known as the hindgut, harbor predominantly *Firmicutes* and *Bacteroidetes*, like the rumen, but with different dominant genera such as *Bacteroides*, *Clostridium*, and *Ruminococcus* [6,7]. The hindgut serves as a secondary fermentation chamber where undigested carbohydrates and other substrates are fermented, producing VFAs, vitamins and amino acids that are absorbed and utilized by the host [8,9,10]. Additionally, the hindgut’s microbial community plays a crucial role in maintaining gut health and preventing pathogenic colonization [9,11,12].

The assembly of microbial communities, shaped through natural selection and coevolution with the host, is influenced by both genetic background and environmental factors [4,7,13]. Different breeds of ruminants possess distinct gastrointestinal microbial communities, as demonstrated by the varying abundance of gastrointestinal microbes in yaks and cattle, which is partly influenced by the host’s adaptability to extreme environments [14]. The Buffalo demonstrated superior fiber digestion compared to the Holstein under identical controlled dietary conditions, which can be attributed to the influence of microbial regulation of the gastrointestinal tract [7]. In a comparable study, Floridia et al. demonstrated that Modicana cows exhibited superior adaptation to olive cake in comparison to Holstein when olive cake was incorporated into the same dietary regimen [15].

The host-gastrointestinal microbial community interaction is a significant factor influencing the differences in ruminant performance. For example, it has been shown that interactions between the host and rumen community affect lactation performance in dairy cows, with the total amount and proportion of rumen microbial fermentation products influencing milk synthesis [16]. Furthermore, it has been proposed that differences in rumen microbial communities, to some extent, account for the observed variations in lactation performance between Jersey and Holstein cows [17]. And a lower abundance of Bacteroidetes has been observed in rumen microorganisms of cows with high milk protein yield compared to cows with low milk protein yield [18].

However, there is currently no research literature on the gastrointestinal microbial communities of Jersey and Holstein dairy cows, and most of the above studies have not explored the differences in the gastrointestinal microbiomes between breeds. Therefore, evaluating the holistic differences in both rumen and hindgut bacterial microbiota between breeds is critical for optimizing health, productivity and overall animal welfare. In the present study, we evaluated the composition of rumen and hindgut bacterial microbiota in a large cohort of two breeds of dairy cows, comprising 184 Jersey cows and 165 Holstein cows. Our objectives were to identify characteristic microbial markers for the rumen and hindgut as well as breed-specific microbial markers for Jersey and Holstein cattle, thereby providing new insights into the mechanisms underlying the differences in production traits between Holstein and Jersey cattle from the perspective of gastrointestinal microorganisms.

## 2. Materials and Methods

### 2.1. Animals and Sample Collection

The experimental animals were 165 Holstein and 184 Jersey cows on a commercial farm in North China, all in first parity and at days in milk (DIM) from 90 to 122 days, with no record of disease or risk of disease during the three-month period. Samples were collected in two batches, October 2020 (50 Holsteins and 81 Jersey cows) and July 2021 of the following year (115 Holsteins and 103 Jersey cows). All cows were fed ad libitum with the same total mixed ration diet for over 2 weeks, and the feed ration composition information is listed in Table S1. Both batches of experimental cows were kept under essentially the same conditions, with the Holstein and Jersey cows housed in completely contiguous barns. The cows were fed three times daily at 0700, 1330, and 1930 h and had free access to water. Sample collection started in the morning after feeding. Rumen liquid was collected using a flexible stomach tube, which was washed with clean water before each collection. About 50 mL of rumen liquid from each cow was aspired through the mouth, with the initial 100 mL (approximately) discarded to avoid contamination by saliva. Hindgut contents were collected from cows using sterile long-walled gloves (a new long-walled gloves was used each time). All samples were dispensed into 5 mL sterile freezing tubes and immediately stored in a liquid nitrogen tank until DNA extraction.

### 2.2. DNA Extraction and 16S rRNA Gene Sequencing

Microbial DNA was extracted from the rumen liquid and hindgut contents of each animal using the E.Z.N.A.^®^ Soil DNA Kit (Omega Biotek, Norcross, GA, USA) in accordance with the manufacturer’s instructions. The final DNA concentration and purity were determined using a NanoDrop 2000 UV-vis spectrophotometer (Thermo Scientific, Wilmington, SC, USA), and the DNA quality was assessed via 1% agarose gel electrophoresis. The V3–V4 hypervariable regions of the 16S rRNA genes were amplified with the primers 338F (5′-ACTCCTACGGGAGGCAGCAG-3′) and 806R (5′-GGACTACHVGGGTWTCTAAT-3′) using a thermocycler PCR system (GeneAmp 9700, ABI, New York, NY, USA).

The polymerase chain reaction (PCR) reactions were conducted using the following program: 3 min of denaturation at 95 °C, 27 cycles of 30 s at 95 °C, 30 s for annealing at 55 °C, 45 s for elongation at 72 °C, and a final extension at 72 °C for 10 min. The PCR reactions were conducted in triplicate using a 20 µL mixture containing 4 µL of 5 × FastPfu Buffer, 2 µL of 2.5 mM dNTPs, 0.8 µL of each primer (5 µM), 0.4 µL of FastPfu Polymerase, and 10 ng of template DNA. The resulting PCR products were extracted from a 2% agarose gel, further purified using the AxyPrep DNA Gel Extraction Kit (Axygen Biosciences, Union City, CA, USA), and quantified using QuantiFluor™-ST (Promega, Madison, WI, USA) according to the manufacturer’s protocol. The construction of a library utilized the NEXTFLEX Rapid DNA-Seq Kit. Finally, the purified amplicons were pooled in equimolar quantities and subjected to paired-end sequencing (2 × 300 bp) on an Illumina MiSeq platform (Illumina, San Diego, CA, USA) in accordance with standard protocols.

### 2.3. Sequencing Data Analysis

The raw amplicon sequencing data were imported into the QIIME 2 software platform (v2023.9) [19] for sequence processing. The dada2 [20] plugin was applied to denoise and dereplicate pair-end sequences, and to filter chimeras. Default values of dada2 arguments were used except for truncating (296 and 210 for forward sequences and reverse sequences, respectively) and trimming sequences (26, including 6 nt barcode and 20 nt primer sequence). Finally, a total of 15,525,818 sequences of 23,613 amplicon sequence variants (ASVs) were obtained.

The phylogenetic tree, diversity indices, and taxonomy were obtained using the phylogeny [21], diversity, and feature-classifier [22] plugins of QIIME 2, respectively. The α-diversity indices included the Observed features, Shannon, Evenness and Faith PD indices, and the β-diversity indices are based on the Bray–Curtis distance. The ASV species classifier training utilized the SILVA SSU reference database (v138) [23] for species annotation, with reference sequences cut according to the V3–V4 fragments of the 16S rRNA genes used in this study.

### 2.4. Bacterial Microbiota Diversity Analysis

In this study, the α-diversity of microbiota was compared between different sampling sites (rumen vs. hindgut) and between different breeds (Holstein vs. Jersey). A linear mixed model (Model 1) was used to perform a differential analysis of α-diversity of rumen or hindgut sample microbiota between species. The beta diversities of microbiota were analyzed using hierarchical clustering and principal coordinate analysis (PCoA). The effects of breed (Jersey vs. Holstein), site (hindgut vs. Rumen), and lactation stage (DIM 90 to 99d vs. DIM 100 to 122) on bacterial microbiota beta diversities were tested using a permutation multivariate ANOVA (PERMANOVA) with a permutation number of 999.

$$y_{ijklm}=\mu +B_{i}+S_{j}+B_{i}\times S_{j}+T_{k}+C_{l}+e_{ijklm}\;(\mathrm{Model}\;1)$$

where $y_{ijklm}$ is the α-diversity index; *μ* is the population mean; $B_{i}$ is the breed effect; *i* = 1 to 2; $S_{j}$ is the sampling site effect; *j* = 1 to 2; $B_{i}\times S_{j}$ is the breed and sampling site interaction effect; $T_{k}$ is the random effect of sampling batch; *k* = 1 to 2; $C_{l}$ is the random effect of cows; and $e_{ijklm}$ is the random error.

### 2.5. Differential Bacterial Microbiota Analysis

A differential microbiological analysis was conducted using the linear discriminant analysis effect size (LEfSe) [24] to identify gastrointestinal microbiological markers across sites (rumen vs. hindgut) and breeds (Holstein vs. Jersey). Subclasses were created based on sampling batches to identify consistent microbiological markers across batches. Microbiological markers of the rumen and hindgut bacterial microbiota were analyzed separately for both breeds and the results were then compared between two breeds to identify any consistent rumen and hindgut microbiological markers between the breeds. The steps of the LEfSe analysis were as follows:

Step 1: Kruskal–Wallis (KW) test between classes (Table 1). Differences in bacterial microbiota components between classes were examined using KW test and microbial species were screened for significant differences (FDR < 0.01).

Step 2: Wilcoxon test between subclasses (Table 1). For microbial samples that showed significant differences between classes in step 1, the Wilcoxon test was performed on each batch in different classes to identify species that were significantly different between all subclasses (FDR < 0.01) and where the direction of the difference was consistent with the KW test between classes.

Step 3: Linear discriminant analysis (LDA). For the different microorganisms screened in Step 2, an LDA was conducted between classes to obtain the LDA score (log10(LDA score)) for each species. Species with a log10(LDA score) > 3 were identified as microbial markers.

## 3. Results

### 3.1. Differences in the Rumen and Hindgut Microbiota

**Figure 1:**
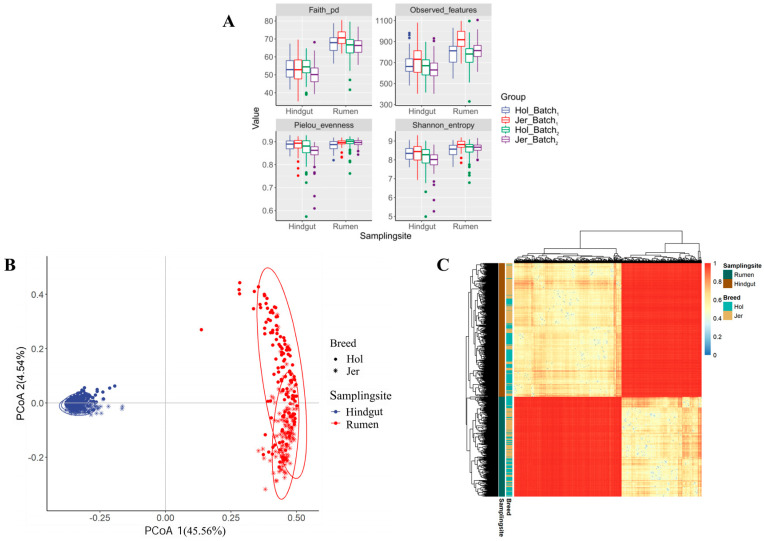
Comparison of the microbial diversity between rumen and hindgut in Holstein (Hol) and Jersey (Jer) cows. (**A**) Alpha diversity of rumen and hindgut microbiota; (**B**) PCoA based on Bray–Curtis distance; (**C**) Hierarchical clustering based on Bray–Curtis distance.

Interactions between breeds and GIT location (hindgut vs. rumen) on bacterial microbiota were observed. As shown in Figure 1, the α-diversity index of the rumen bacterial microbiota was found to be higher than that of the hindgut sample bacterial microbiota (*p* < 0.01), and the observed differences exhibited a consistent pattern across breeds. The β-diversity analysis of the rumen and hindgut sample bacterial microbiota is presented in Figure 1B,C. The clusters of rumen and hindgut bacterial microbiota were significantly separated from in the first axis of PCoA which represented 45.56% of total variation between microbial communities in terms of Bray–Curtis distance (Figure 1B). Similarly, hierarchical clustering based on the Bray–Curtis distance revealed that the main difference between the clusters was the sampling location (Figure 1C). The differences in bacterial microbiota composition between rumen and hindgut were significant (*p* < 0.01), as demonstrated by a permutation multivariate analysis of variance (PERMANOVA) (Table 2) based on the Bray–Curtis distance. The sampling site exhibited the largest R^2^ among various influencing factors. Furthermore, both the breed and batch had significant effects (*p* < 0.01) on the overall species composition and abundance of the microbiota.

**Figure 2:**
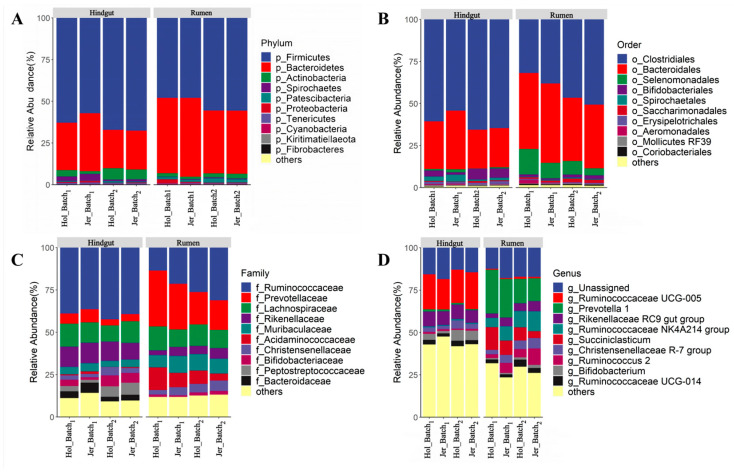
The taxonomic profile of microbial communities at the phylum level (**A**), order level (**B**), family level (**C**) and genus level (**D**) in Holstein (Hol) and Jersey (Jer) cows of GIT. Taxa with relative abundance ranked below 10 were grouped in others.

The taxonomic composition of the rumen and hindgut bacterial microbiota at the taxonomic level is shown in Figure 2. At the phylum level, the dominant phyla in both the rumen and hindgut were *Firmicutes* and *Bacteroides*. The relative abundance of *Bacteroides* was greater in the rumen (41%) than that in the hindgut (27%), whereas the relative abundance of *Firmicutes* was greater in the hindgut (64% and 53% in hindgut and rumen, respectively). The dominant orders of *Firmicutes* were *Clostridia* and *Bacteroidales*. *Bifidobacteriales*, a member of the *Actinobacteria* phylum, exhibited a high relative abundance in hindgut (5%) but a low relative abundance in the rumen (2%). At the family and genus levels, *Prevotellaceae* was predominant in the rumen and were primarily composed of the genus *Prevotella 1*, the most prevalent genus in the rumen. The family *Ruminococcaceae* exhibited a high relative abundance in both the rumen and hindgut. The genus *Ruminococcaceae UCG-005* was the dominant genus in the hindgut, while the genus *Ruminococcaceae NK4A214 group* and the genus *Ruminococcus 2* were mainly found in the rumen.

**Figure 3:**
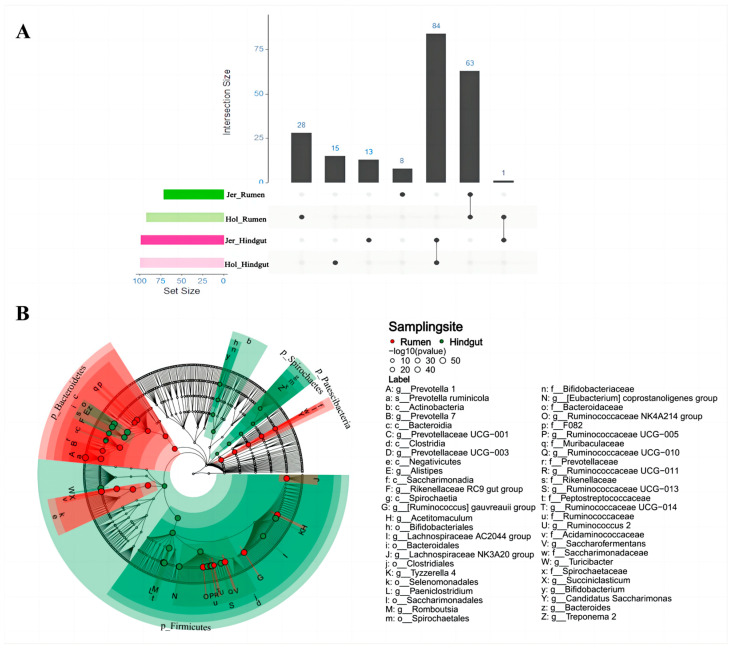
Analysis of microbiological differences between the rumen and hindgut. (**A**) Number of differential microorganisms in rumen and hindgut of Holstein and Jersey cows; (**B**) differential microorganisms of the rumen and hindgut microbiota.

The distribution of rumen and hindgut microbial markers in the two breeds is shown in Figure 3A. A total of 191 and 169 different microorganisms of the rumen and hindgut bacterial microbiota were found in Holstein and Jersey cows, respectively, and the consistency of the different microorganisms of the rumen and hindgut bacterial microbiota in the two breeds of dairy cows was high, with 63 out of the 100 microbial markers of the rumen being consistent between breeds and 84 out of the 113 hindgut microbial markers being consistent between breeds.

Differential microorganisms in the rumen and hindgut was shown in Figure 3B. The distribution of rumen microbial markers was mainly observed in *Bacteroidetes*, including *Bacteroidia*, *Bacteroidales*, *Bacteroidaceae*, and *Prevotella*, whereas their relative abundance was very low in the hindgut. The rumen microbial markers also include bacteria belonging to other phyla with high relative abundances, such as bacterial genus *Succiniclasticum* and *Ruminococcaceae NK4A214 group* belonging to *Firmicutes*. The microbial markers in hindgut were found to be mainly present in *Firmicutes*, including *Clostridiales* and *Ruminococcaceae*. Additionally, differential microorganisms belonging to the *Spirochaetes* were enriched in hindgut, such as bacterial class *Spirochaetia* and bacterial genus *Treponema 2*.

### 3.2. Differences in the Rumen Bacterial Microbiota Between Holstein and Jersey Cows

**Figure 4:**
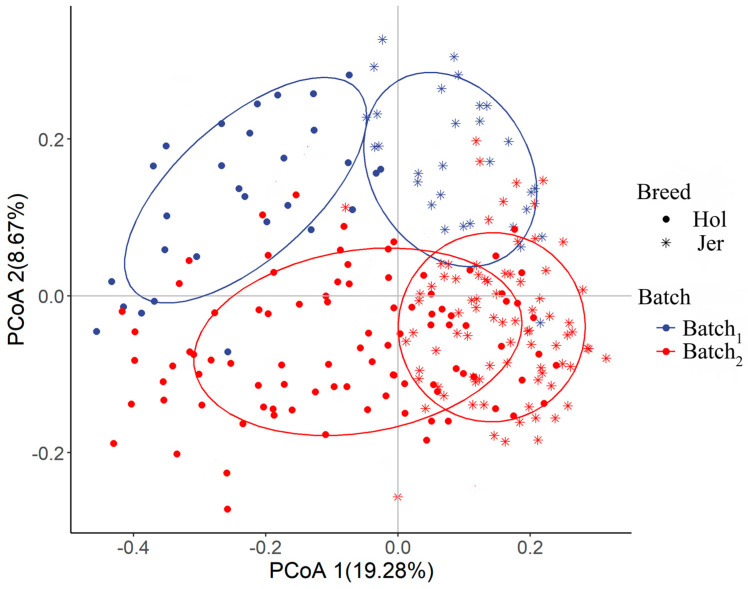
Comparison of rumen microbial diversity between the Holstein (Hol) and Jersey (Jer) cows.

The PCoA of rumen bacterial microbiota based on the Bray–Curtis distance showed two separated clusters of samples from Holstein and Jersey in the first principal coordinate (Figure 4). As shown in Table 3, the effect of breed, batch and their interaction on rumen bacterial microbiota was significant revealed by PERMANOVA test using the Bray–Curtis distance (*p* < 0.01).

**Figure 5:**
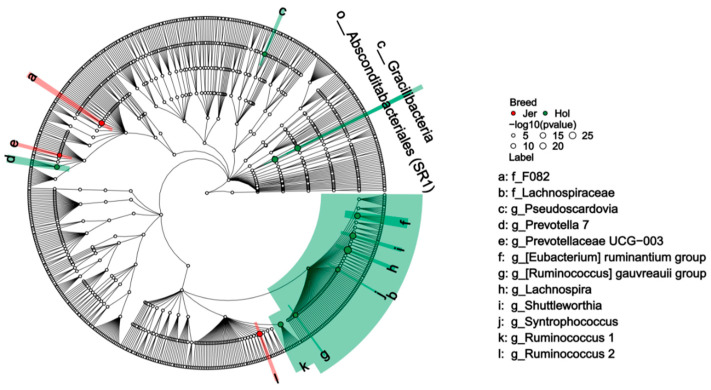
Differences in the rumen bacterial microbiota between the Holstein (Hol) and Jersey (Jer) cows.

A screening of rumen microbial markers that were differentially abundant in two breeds, with consistency across batches conducted using LEfSe. A total of 14 differentially abundance microbes with taxonomic annotations were identified between the two breeds, with 11 differential microbes enriched in the rumen of Holstein and 3 microbes enriched in Jersey cows (Figure 5). Three differential rumen microbes enriched in Jersey cows were F082, Ruminococcus 2 and Prevotellaceae UCG-003. Notably, six genera of the *Lachnospiraceae* family, including *[Eubacterium] ruminantium group*, *[Ruminococcus] gauvreauii group*, *Lachnospira*, *Shuttleworthia* and *Syntrophococcus*, as well as the family itself, were enriched in the rumen of Holstein cows. Three genera, *Pseudoscardovia*, *Ruminococcus 1*, and *Prevotella 7*, as well as the class *Gracilibacteria*, were also enriched in the rumen of Holstein cows.

### 3.3. Differences in Hindgut Bacterial Microbiota Between Holstein and Jersey Cows

**Figure 6:**
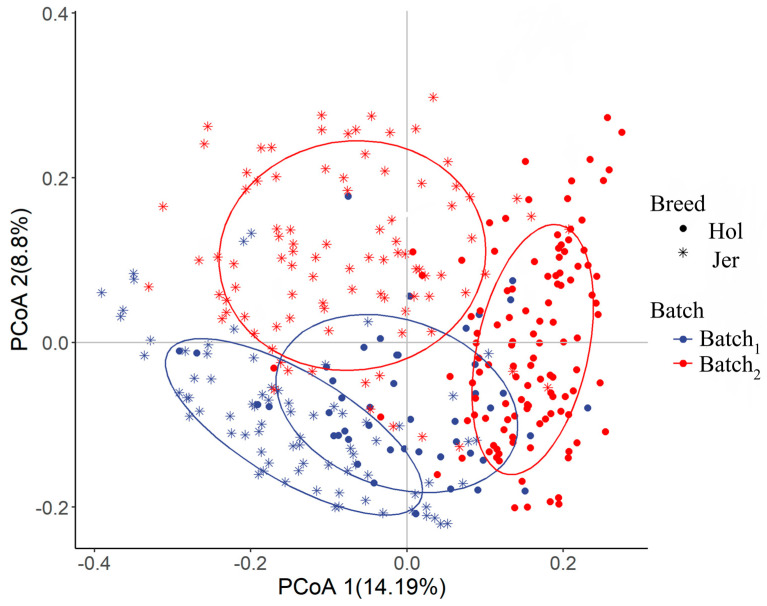
Comparison of hindgut microbial diversity between the Holstein (Hol) and Jersey (Jer) cows.

The PCoA based on Bray–Curtis distances showed that the hindgut bacterial microbiota was significantly different between Holstein and Jersey cows in the first principal axis (Figure 6). The results of the PERMANOVA test indicated that there was a highly significant effect of batch and breed, as well as of the interactions between the batch and breed on the composition of the bacterial microbiota (*p* < 0.01, Table 4). Among the effects, breed explained the greatest proportion of the overall variation in hindgut microbiota.

**Figure 7:**
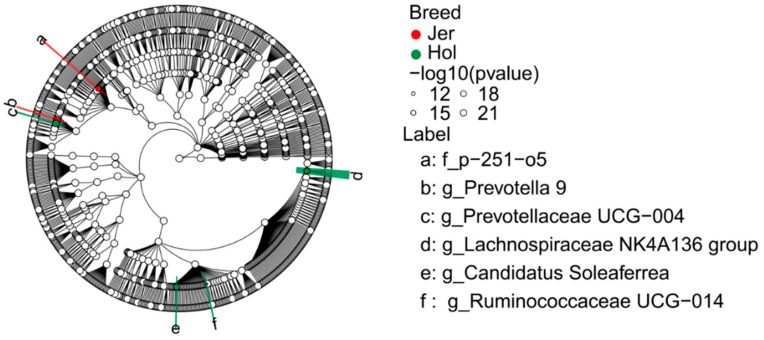
Differences in the hindgut bacterial microbiota between the Holstein (Hol) and Jersey (Jer) cows.

A rigorous screening for hindgut microbial markers between breeds with consistent patterns in different batcher was conducted using LEfSe. A total of 13 different hindgut microbes were identified between the breeds, 6 of which had clear taxonomic annotations, including 4 hindgut microbial markers from Holstein cows and 2 from Jersey cows. The distribution of hindgut microbial markers among species at each taxonomic level is illustrated in Figure 7. The family *p-251-o5* and the genus *Prevotella 9* were identified as hindgut microbial markers of Jersey cows, while *Prevotellaceae UCG-004*, *Lachnospiraceae NK4A136 group*, *Candidatus Soleaferrea* and *Ruminococcaceae UCG-014* were identified as hindgut microbial markers of Holstein cows.

## 4. Discussion

### 4.1. Spatial Characteristics of Microbiota

The overall composition of bacterial communities in the rumen and hindgut showed significant discrepancies regardless of cow breeds, as revealed by both α- and β-diversity analysis. In terms of α-diversity, the rumen bacterial microbiota exhibited higher species richness than the hindgut, which is consistent with other studies on calves [25], beef cattle [26], dairy cows [27] and buffaloes [7]. The higher species richness in the rumen may be attributed to the greater availability of substrates for microbial fermentation, which account for approximately 50% of the entire gastrointestinal nutrients of ruminants [27]. Furthermore, when the gastrointestinal contents are transferred from the stomach to the hindgut intestine for secondary fermentation, some microorganisms attached to the surface of digesta are absorbed by the host, resulting in a reduction in overall species richness in the hindgut [10,28].

However, it was also observed in the current study that the differences in the composition of the rumen and hindgut bacterial microbiota were consistent between the Jersey and Holstein cows. The consistency between two cattle breeds may result from convergent evolution, where cattle develop similar gut structures for herbivory, leading their bacterial microbiota to arrive at similar compositional configurations in different breeds with similar gut structures [28]. The differences between gastrointestinal sites may be attributed to variations in substrate availability, the retention time of digesta, and pH levels at various sites in the GIT of ruminants [13], which create distinct spatial characteristics of the GIT and have a profound impact on local microbial assemblages and functions [12].

The rumen and hindgut have distinct core microbiota. In the rumen, *Prevotella* of the phylum *Bacteroidetes* was the most abundant genus, displaying a higher relative abundance compared to the hindgut. This observation aligns with findings from other studies on the gastrointestinal bacterial microbiota of ruminants [7,13]. *Prevotella* are capable of fermenting a variety of carbohydrates, including starch, and the rumen provides more readily fermentable substrates than the hindgut [9,27], creating the optimal conditions for the growth and proliferation of *Prevotella*. Furthermore, *Prevotella* serves as a source of microbial protein that is degraded and absorbed by the host in the foregut, which may contribute to its relatively low abundance in the hindgut [10]. The predominant bacteria in the hindgut primarily consisted of *Ruminococcus* from the phylum *Firmicutes*. *Ruminococcus* is highly effective at degrading fiber and can adhere in large quantities to plant fibers, continuing to ferment incompletely digested fibers as they are transported to the hindgut [1]. *Ruminococcus* has the lipopolysaccharides or lipoteichoic acids in its cell wall provide a certain level of protection [10], reducing degradation and absorption in the foregut during transportation. This partly explains the high relative abundance of *Ruminococcus* in the hindgut.

### 4.2. Differences in Gastrointestinal Bacterial Microbiota Among Breeds

Despite the similarity between different ruminant breeds, an individual’s life trajectory and host genetics also exert a significant influence on the microbial communities. In this study, it was found that there were significant differences in the dominant bacterial microbiota in the rumen and hindgut between Holstein and Jersey cows. The bacteria that were significantly abundant in the Holstein cows mostly belong to *Firmicutes*, while those predominantly found in the Jersey cows mostly belong to *Bacteroidetes*. This finding is consistent with a previous study assessing the differences in rumen bacterial microbiota between Jersey and Holstein cows [17]. The gastrointestinal microbiome significantly influences the productivity of ruminants. The evidence shows that an increase in the ratio of *Firmicutes* to *Bacteroidetes* (F/B ratio) is associated with improved feed conversion rates in the host [29,30]. The difference in the relative abundance of *Firmicutes* and *Bacteroidetes* between Holstein and Jersey cows may attribute to differences in the production performance between two breeds, which is indicated by the association of the F/B ratio with feed efficiency [31,32].

The findings of this study indicate that members of the *Lachnospiraceae* and *Ruminalococcus*, which belong to the phylum *Firmicutes*, were present at significantly higher levels in the Holstein cow population than in the Jersey cow population. Additionally, the same bacteria were found to be significantly more abundant in individuals with high feed efficiency, whereas the *Prevotella* was present in both efficient and inefficient individuals [13,33]. And it was also demonstrated that individuals with inefficient feed possess a more diverse gastrointestinal microbiome, demonstrating the ability to ferment a wider range of substrates and produce a greater number of products. However, these products can be deleterious, of little or no benefit, or exceed the host’s absorption capacity (as determined by host genetics), leading to feed inefficiency. Conversely, efficient cattle with a relatively simple gastrointestinal microbiome may produce more specific products that can be absorbed and utilized more efficiently by the host [32]. This partly explains the higher observed features found in the present study in Jersey cows than in Holstein cows. Considering all of the above combined with the fact that Holsteins have a higher feed conversion rate than Jersey cows [34], we hypothesize that breed-specific microorganisms may play a key role in shaping the differences in performance between the two breeds of dairy cows.

## 5. Conclusions

In summary, our study demonstrated that the bacterial communities in the rumen and hindgut of Jersey and Holstein cows exhibited notable differences in composition and abundance, with the rumen displaying significantly higher bacterial microbial diversity than the hindgut. A total of 14 rumen bacteria and 6 hindgut bacteria were identified as breed-differentiated bacteria, they may contribute to the differences in lactation performance between the Jersey and Holstein cows. These findings provide new insights into the mechanisms underlying differences in production traits between Holstein and Jersey cattle from a gastrointestinal microbiological perspective.

## Figures and Tables

**Table 1:** Classing and subclass setting in LEfSe analysis.

Analyzed Content	Classes	Subclasses
Microbiological markers of rumen and hindgut microbiota	Rumen	Batch1
		Batch2
	Hindgut	Batch1
		Batch2
Microbiological markers of rumen and hindgut sample bacterial microbiota between breeds	Holstein	Batch1
		Batch2
	Jersey	Batch1
		Batch2

**Table 2:** The PERMANOVA analysis of the Bray–Curtis distance based on the gastrointestinal bacterial microbiota of Jersey and Holstein cows.

	Df	Sum of Sqs	R^2^	F	*p* Value
Sampling site	1	87.32	0.45	520.26	0.001
Batch	1	3.26	0.02	19.41	0.001
Breed	1	3.87	0.02	23.04	0.001
Lactation stage	1	0.50	0.00	3.01	0.072
Residual	587	98.52	0.51		
Total	591	193.47	1		

**Table 3:** The PERMANOVA analysis of the Bray–Curtis distance based on the rumen bacterial microbiota of Jersey and Holstein cows.

	Df	SumOfSqs	R^2^	F	*p* Value
Breed	1	3.45	0.08	22.16	0.001
Batch	1	2.69	0.06	17.27	0.001
Breed:Batch	1	0.73	0.02	4.67	0.001
Residual	249	38.77	0.85		
Total	252	45.64	1		

**Table 4:** The PERMANOVA analysis of the Bray–Curtis distance based on the hindgut bacterial microbiota of Jersey and Holstein cows.

	Df	SumOfSqs	R^2^	F	*p* Value
Breed	1	4.21	0.07	27.09	0.001
Batch	1	3.13	0.05	20.12	0.001
Breed:Batch	1	1.06	0.01	6.82	0.001
Residual	335	52.10	0.86		
Total	338	60.51	1		

## Data Availability

The datasets used during the current study are available from the corresponding author on reasonable request.

## Supplementary Materials

The following supporting information can be downloaded at: https://www.mdpi.com/article/10.3390/ani14213137/s1. Table S1: Composition of diets for Jersey and Holstein cows.
